# Case report: Hereditary spastic paraplegia with a novel homozygous mutation in *ZFYVE26*

**DOI:** 10.3389/fneur.2023.1160110

**Published:** 2023-08-23

**Authors:** Ze-hua Lai, Xiao-ying Liu, Yuan-yue Song, Hai-yan Zhou, Li-li Zeng

**Affiliations:** ^1^Department of Neurology and Institute of Neurology, Ruijin Hospital, Shanghai Jiao Tong University School of Medicine, Shanghai, China; ^2^Department of Neurology, Yangpu Hospital, Tongji University School of Medicine, Shanghai, China

**Keywords:** hereditary spastic paraplegia, *ZFYVE26*, case report, novel homozygous mutation, SPG15

## Abstract

Hereditary spastic paraplegia (HSP) is a group of neurodegenerative diseases with genetic and clinical heterogeneity characterized by spasticity and weakness of the lower limbs. It includes four genetic inheritance forms: autosomal dominant inheritance (AD), autosomal recessive inheritance (AR), X-linked inheritance, and mitochondrial inheritance. To date, more than 82 gene loci have been found to cause HSP, and SPG15 (*ZFYVE26*) is one of the most common autosomal recessive hereditary spastic paraplegias (ARHSPs) with a thin corpus callosum (TCC), presents with early cognitive impairment and slowly progressive leg weakness. Here, we reported a homozygous pathogenic variant in *ZFYVE26*. A 19-year-old Chinese girl was admitted to our hospital presenting with a 2-year progressive bilateral leg spasticity and weakness; early cognitive impairment; corpus callosum dysplasia; chronic neurogenic injury of the medulla oblongata supplied muscles; and bilateral upper and lower limbs on electromyogram (EMG). Based on these clinical and electrophysiological features, HSP was suspected. Exome sequencing of the family was performed by high-throughput sequencing, and an analysis of the patient showed a *ZFYVE26* NM_015346: c.7111dupA p.(M2371Nfs^*^51) homozygous mutation. This case reported a new *ZFYVE26* pathogenic variant, which was different from the SPG15 gene mutation reported earlier.

## Introduction

Hereditary spastic paraplegia (HSP), also called spastic paraplegia (SPG), is a group of neurodegenerative diseases with genetic and clinical heterogeneity characterized by spasticity and weakness of the lower limbs (1), and the prevalence is ~1.8/100,000 (2). It includes four genetic inheritance forms, namely: autosomal dominant inheritance (AD), autosomal recessive inheritance (AR), X-linked inheritance, and mitochondrial inheritance (2). Until now, over 82 gene loci have been found to cause HSP (3–5). HSP patients may have either pure or complicated HSP, differing based on symptoms. Patients with pure HSP simply develop spasticity and weakness of the lower limbs (6), while patients with complicated HSP are often accompanied by other symptoms, such as early cognitive impairment, ataxia, visual disturbance, macular degeneration, dysarthria, and callosal agenesis (7).

SPG15 (Spastic Paraplegia type of 15, *ZFYVE26*) is one of the most common ARHSPs with thin corpus callosum (TCC) (8), and it presents with early cognitive impairment and slowly progressive leg weakness (9). *ZFYVE26* gene is localized at 14p24.1, and it encodes a zinc finger protein with an FYVE domain called “spastizin” (10). It forms a protein complex with Spatacsin (SPG11) and *KIAA0415* (SPG48), and participates in various cellular events such as membrane trafficking and signal transduction (10).

Here, we reported a homozygous mutation in *ZFYVE26*. A 19-year-old Chinese girl was admitted to our hospital presenting with a 2-year progressive bilateral leg spasticity and weakness, and early cognitive impairment; corpus callosum dysplasia and chronic neurogenic injury of the medulla oblongata supplied muscles; and bilateral upper and lower limbs on electromyogram (EMG). Based on these clinical features and the electrophysiological findings, HSP was suspected. Exome sequencing of the family was performed by high-throughput sequencing, and an analysis of the patient showed a c.7111dupA p.(M2371Nfs^*^51) (Exon 38) homozygous novel mutation in *ZFYVE26* gene. This case reported a new *ZFYVE26* pathogenic variant.

## Case presentation

**Figure 1:**
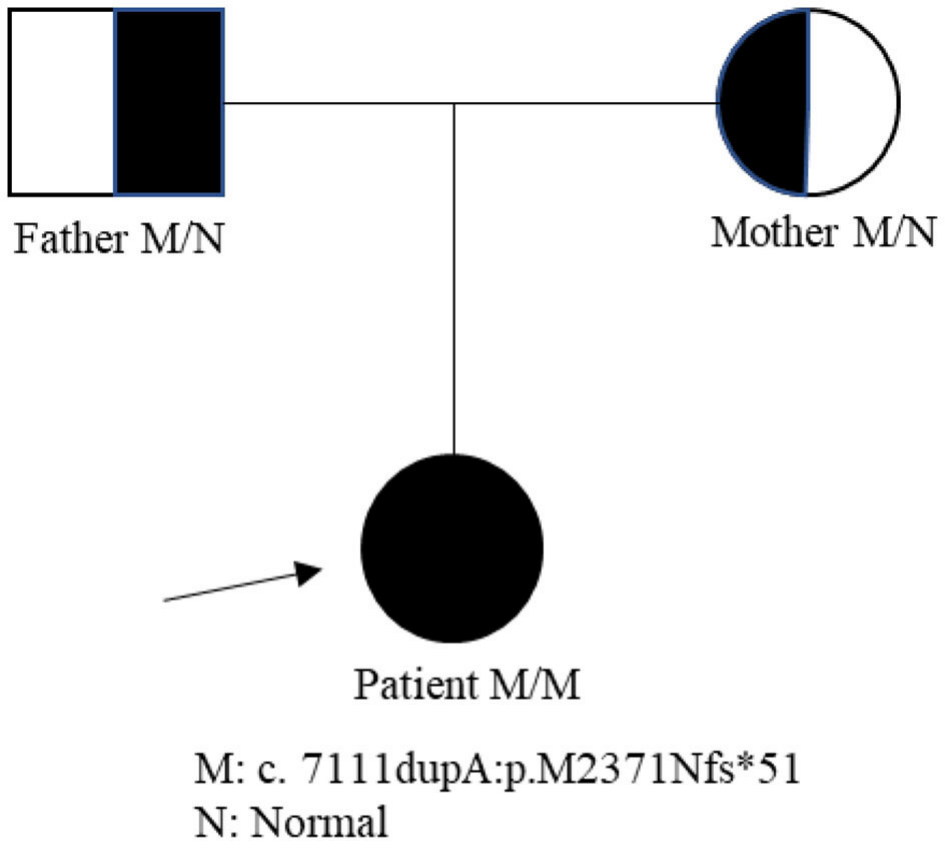
The patient's family tree of the novel mutation *ZFYVE26*.

A 19-year-old girl was admitted to our hospital presenting with a 2-year progressive bilateral leg spasticity and weakness. Her medical history was not remarkable, and her family history was negative for genetic disease. Vital signs were in the normal ranges: body temperature, 36.7°C; respiratory rate, 22 breaths/min; pulse rate, 72 bpm; and blood pressure, 126/68 mm Hg. Neurological examinations revealed that the lower limbs' muscle strengths were of grades 3–5; hypermyotonia in her lower limbs, hyperreflexia in the knee and ankle reflexes, and bilateral Babinski signs (+), Chaddock signs (+), Gordon signs (+), and Oppenheim signs (+). Cranial nerve, the upper limb, and ataxia examination showed no abnormalities. She also had mild mental deficiency with a MoCA score of 22. Lab examinations, including metabolic, tumor marker, and immunity parameters, were all within normal ranges. Brain and cervical MRI revealed corpus callosum dysplasia and cervical disk herniation in C3/4 and C5/6. EMG reported that F wave latency for the ulnar nerve and the tibial nerve was normal; but EMG of the anterior tibial muscle showed fibrillations and positive sharp waves; and there were widened MUP time limit, increased wave amplitude, and increased polyphase wave during the light contraction in most muscles, and decreased recruitment phase during recontraction. These findings indicated that there was extensive chronic neurogenic electromyographic impairment; and anterior horn involvement was considered first (ball, cervix, and lumbar). The diagnosis of HSP was suspected. Biopsy of the right biceps muscle showed no obvious hyperplasia of connective tissue, no abnormalities in muscle bundle of the small vessel wall, and no inflammatory cells infiltration or abnormal deposits around blood vessels; and there was no muscle fiber atrophy, hypertrophy, necrosis, swirl or spiral change, no nuclei aggregation, and no vacuolar formation in muscle fiber. Therefore, neurogenic skeletal muscle injury was suspected. Second-generation gene sequencing test revealed that the patient carried a *ZFYVE26* NM_015346: c.7111dupA p.(M2371Nfs^*^51) homozygous mutation, which came from her heterozygous parents (Figure 1). Combined with clinical symptoms, a diagnosis of SPG15 was made. The patient was given 5 mg of baclofen twice a day. However, there was no significant improvement in her symptoms on discharge, but her symptoms were alleviated gradually in 3 months of follow-up.

## Discussion

HSP, a kind of genetic neurodegenerative disease and clinical heterogeneity characterized by spasticity and weakness of the lower limbs, can be classified into two types, namely, the pure type (clinical manifestations include typical muscle spasms, hyperreflexia, clonus, gait disorder, and bladder dysfunction) (6) and the complicated type (besides the abovementioned symptoms, clinical manifestation includes early cognitive impairment, ataxia, visual disturbance, macular degeneration, dysarthria, and callosal agenesis) (11, 12).

The pathogenesis of HSP remains unknown, and the axonal degeneration caused by the various types of HSP has different molecular pathogenesis. There are four modes of mutation, namely, AD, AR, X-linked inheritance, and mitochondrial inheritance (13). To date, over 82 gene loci have been found to cause HSP (3). These genes are involved in many cellular events such as membrane trafficking, signal transduction, the morphology of the endoplasmic reticulum, microtubule dynamics and transport, mitochondrial function, lipid metabolism, and endosome/lysosome functions. The main pathological change of HSP is axonal degeneration, which may also be accompanied by other changes such as demyelination and loss of neurons. Axonal degeneration of the corticospinal tract (most obvious in the thoracic spinal cord) and fasciculus gracilis fibrosis (most obvious in the cervical spinal cord) have also been detected in autopsy.

SPG15 (*ZFYVE26*) is a kind of early-onset complex ARHSP, which is characterized by typical atrophy of the corpus callosum, and its main clinical symptoms include urinary urgency and incontinence, visual impairment, retinal and macular degeneration, nystagmus, mood fluctuation, mental impairment, ataxia, spastic paraplegia, dysarthria, arcus plantaris, lower limb spasm, callosal agenesis, clonicity, fecal incontinence, bladder sphincter dysfunction, peripheral axon neurodegeneration, distal muscle atrophy, and lower limb muscle weakness (9). Most patients with SPG15 had the first symptoms in their adolescence, some born to consanguineous family even had language delay in infancy and gait disorder at the age of 11 years (5). Our patient presented with classical complicated type of HSP symptoms, including the paralysis of motor neurons in both lower limbs, mild mental impairment, and callosal agenesis. She had progressive bilateral leg spasticity and weakness at the age of 17 years but had no visual or hearing impairment. However, 19 disease-related genes caused HSP manifesting progressive spasticity of the lower limbs and TCC, including SPG1, SPG11, SPG15, SPG21, SPG30, SPG32, SPG35, SPG44, SPG44(65), SPG46, SPG47, SPG48, SPG49, SPG50, SPG52, SPG54, SPG56, SPG63, and SPG71. Furthermore, the onset of these SPGs occurs mainly in children and adolescents and is often accompanied by intellectual disability. While it is hard to distinguish them from clinical symptoms, gene testing is good at detecting specific genetic mutations. Second-generation gene sequencing test proved that this patient had a *ZFYVE26* NM_015346: c.7111dupA p.(M2371Nfs^*^51) homozygous mutation, and both her parents carried a *ZFYVE26* NM_015346: c.7111dupA p.(M2371Nfs^*^51) heterozygous variant. However, this gene loci mutation had not been reported earlier. Therefore, the patient was diagnosed with SPG15, which was composed of AR.

*ZFYVE26*/Spastizin mutations increase immature autophagosomes and lead to autophagy defects. A complex that is composed of *ZFYVE26*/Spastizin, SPG11/Spatacsin and AP5 (adaptor-related protein complex 5) is important in autophagic lysosomal reformation (ALR) (10). Although both *ZFYVE26* and SPG11 interact with RAB5A and RAB11, the two proteins regulating endosome trafficking and maturation, only *ZFYVE26* mutations affect RAB protein interactions and activation and make the fusion between autophagosomes and endosomes defective (10). The *ZFYVE26* c.7111dupA p.M2371Nfs^*^51 mutation identified in our patient is a novel mutation. This frameshift mutation possibly leads to the loss of some amino acid residues in the final protein product and causes loss of function. To date, there have been 62 mutations reported for SPG15 (Table 1) (5, 14–18, 21, 25, 26, 29, 31–38). There are 62.90% (39/62) missense mutations, 29.03% (18/62) deletion mutations, 3.23% (2/62) duplication mutations, and 8.06% (5/62) insertion mutations (Table 1). We searched published literature in PubMed and Web of Science databases and analyzed the clinical features of these 62 kinds of *ZFYVE26* mutations in 84 patients (Table 1). Most patients first had walking gait disorder at young age (13.75 ± 7.85 years), and some had language delay in infancy. The main clinical characteristics of the patients with these mutations include low limbs weakness (98.81%), spasticity (96.43%), and amyotrophy (30.95%); cognitive impairment (39.29%), mental abnormality (50%), dysarthria (46.43%), TCC (47.62%), and white matter hyperintensities (WMH) (39.29%) on MRI; axonal peripheral polyneuropathy (35.71%), Babinski sign positive (27.39%), urinary problems (21.43%), and sphincter (5.95%); and ataxia (17.86%), retinal degeneration (19.05%), nystagmus (14.29%), clawfoot (13.10%), strephenopodia (1.19%), sensory abnormalities, action tremor (9), epilepsy (10.71%), hearing impairment (5.95%), upper limbs weakness (7.14%), spasticity (10.71%), and amyotrophy (5.95%).

**Table 1:** SPG15 gene loci mutation and clinical features.

**Location**	**Mutation**	**References**	**Sex**	**Age**	**Age at onset**	**Lower limb weakness/ spasticity/ amyotrophy**	**Cognitive impairment/ Mental abnormality**	**Dysarthria**	**Axonal peripheral polyneuropathy**	**Babinski sign**	**Urinary problems/ Sphincter disturbances**	**Ataxia**	**Retinal degeneration/ Nystagmus**	**Clawfoot/ Strephenopodia**	**Sensory abnormalities decreased vibration sense; Superficial sensory; Deep sensory**	**Action tremor**	**Epilepsy**	**Hearing impairement**	**Upper limb weakness/ spasticity/ amyotrophy**	**MRI signs**			
																				**TCC**	**WMH**	**Cortical atrophy**	**Prominent cerebellar atrophy**
Exon 2	c.43C > T p.Q15^*^	Goizet et al. (14)	NA	NA	NA	NA	NA	NA	NA	NA	NA	NA	NA	NA	NA	NA	NA	NA	NA	NA	NA	NA	NA
Intron 3-4	c.273 + 8G > A	Yoon et al. (15)	M	18	3	+/+/+	-/-	+	-	-	-/-	-	-/-	-/-	-/-/-	-	-	-	-/-/-	+	-	-	-
Exon 4	c.307G > T p.E103^*^	Goizet et al. (14)	F	41	7	+/+/-	+/+	+	+	+	+/-	-	+/+	-/-	-/-/-	-	-	-	-/-/-	+	+	-	-
Exon 5	c.427G > T p.E143^*^	Goizet et al. (14)	F	41	7	+/+/-	+/+	+	+	+	+/-	-	+/+	-/-	-/-/-	-	-	-	-/-/-	+	+	-	-
	c.592C > T p.R198^*^	Schüle et al. (16)	M	19	17	+/+/-	+/+	+	-	-	-/-	-	-/-	-/-	-/-/-	-	-	-	-/-/-	+	+	+	-
	c.728T > C p.L243P	Vantaggiato et al. (17)	NA	55	38	-/+/-	-/+	+	-	+	+/-	-	-/-	-/-	-/+/-	-	-	-	-/-/-	+	+	-	-
			NA	51	34	-/+/-	-/+	+	-	+	+/-	-	-/-	-/-	-/+/-	-	-	-	-/-/-	+	+	-	-
	c.836T > G		F	15	13	+/+/-	-/-	-	-	-	+/-	-	-/-	-/-	-/-/-	-	-	-	-/-/-	-	+	-	-
Exon 8	c.1240G > T p.E414^*^	Goizet et al. (14)	F	31	13	+/+/+	-/+	-	-	+	+/-	-	-/+	-/-	-/-/-	+	+	-	-/-/-	-	-	-	-
Exon 10	c.1471C > T p.Q491^*^	Pensato et al. (18)	M	25	NA	+/+/-	-/-	-	-	-	-/-	-	-/-	-/-	-/-/-	-	-	-	-/-/-	-	-	-	-
	c.1477C > T p.Q493^*^	Hanein et al. (19)	F	20	14	+/+/-	-/-	-	-	-	-/-	-	-/-	+/-	-/-/-	-	-	-	-/-/-	-	-	+	+
			M	32	12	+/+/+	-/+	+	+	-	-/-	-	-/-	+/-	-/-/-	-	-	-	-/-/+	-	-	-	-
	c.1523T > A p.I508N	Vantaggiato et al. (17)	NA	46	20	+/+/+	-/+	+	+	+	-/-	-	-/-	+/-	-/+/+	-	-	-	-/+/+	+	+	+	-
			NA	47	22	+/+/+	-/+	+	+	+	-/-	+	-/-	+/-	-/+/+	-	-	-	-/+/+	+	+	-	-
	c.1630_1631 delTC p.S544Lfs^*^ 824	Riazuddin et al. (20)	NA	NA	13	+/+/+	+/-	-	+	-	-/-	-	-/-	-/-	-/-/-	-	-	-	-/-/-	-	+	-	-
Exon 11	c.1730delA + c.1731C > T p.Asn577 IIefs^*^36	Pensato et al. (18)	NA	NA	NA	NA	NA	NA	NA	NA	NA	NA	NA	NA	NA	NA	NA	NA	NA	NA	NA	NA	NA
	c.1792delG p.D599 Tfs^*^163	Denora et al. (21)	F	33	21	+/+/-	-/-	-	-	+	-/-	-	+/-	-/-	-/-/-	-	-	+	-/-/-	-	-	-	-
	c.1844C > T p.S615F	Schüle et al. (16)	NA	NA	16	+/+/-	-/-	+	-	-	-/-	+	-/-	-/-	-/-/-	-	-	-	-/-/-	-	-	+	-
			NA	NA	1	+/+/-	-/+	+	+	-	-/-	+	-/-	-/-	-/-/-	-	-	-	-/-/-	-	-	-	-
	c.1925C > T p.A642V	Schüle et al. (16)	NA	NA	1	+/+/-	-/+	-	-	-	-/-	-	-/-	-/-	-/-/-	-	-	-	-/-/-	-	-	-	-
	c.2049delT p.Phe 683Leufs^*^685	Hanein et al. (19)	F	28	11	+/+/+	-/+	+	+	-	-/-	-	-/-	+/-	-/-/-	-	-	-	-/-/-	-	-	-	-
			F	27	8	+/+/+	-/+	-	-	-	-/-	-	-/-	-/-	-/-/-	-	-	-	-/-/-	-	-	-	-
			F	9	8	+/+/-	-/+	-	-	-	-/-	-	-/-	-/-	-/-/-	-	-	-	-/-/-	-	-	-	-
	c.2074delC p.L692Sfs^*^52	Tunca et al. (22)	NA	NA	17	+/+/-	-/-	-	-	-	-/-	-	-/-	-/-	-/-/-	-	-	-	-/-/-	-	-	-	-
	c.2182C > T p.R728^*^	Goizet et al. (14)	M	17	12	+/+/-	+/+	-	-	-	-/-	-	-/-	-/-	-/-/-	+	-	-	+/+/-	+	+	-	-
	c.2196_2198 delTGT p.V733del	Karakaya et al. (23)	NA	NA	NA	NA	NA	NA	NA	NA	NA	NA	NA	NA	NA	NA	NA	NA	NA	NA	NA	NA	NA
Exon 12	c.2254C > T p.Glu752^*^	Pensato et al. (18)	NA	NA	NA	-/-/-	-/-	-	-	-	-/-	-	-/-	-/-	-/-/-	-	-	-	-/-/-	-	-	-	-
	c.2331_ 2332insA p.D778Rfs^*^15	Goizet et al. (14)	M	17	12	+/+/-	+/+	-	-	-	-/-	-	-/-	-/-	-/-/-	+	-	-	+/+/-	+	+	-	-
	c.2332 + 7delT	Schüle et al. (16)	NA	NA	22	+/+/-	-/-	-	+	-	-/-	-	-/-	-/-	-/-/-	-	-	-	-/-/-	-	-	-	-
Exon 13	c.2338C > T p.R780^*^	Pyle et al. (24)	M	NA	32	+/+/-	-/-	-	+	-	-/-	+	-/-	-/-	-/-/-	-	-	-	-/-/-	-	-	+	-
	c.2450delT p.L817Cfs^*^12	Pyle et al. (24)	M	NA	32	+/+/-	-/-	-	+	-	-/-	+	-/-	-/-	-/-/-	-	-	-	-/-/-	-	-	+	-
Exon 14	c.2554-1 G > A	Renvoisé et al. (25)	F	26	15	+/+/-	+/+	+	-	-	-/-	-	-/-	-/-	-/-/-	+	-	-	-/-/-	+	+	-	-
	c.C2254T p.Q752^*^	Vinci et al. (26)	M	27	18	+/+/+	+/+	-	+	-	+/-	+	-/-	-/-	-/-/-	-	+	-	-/-/-	+	+	+	-
Exon 15	c.2615_2617 delGCTi nsTGAA p.R872 Lfs^*^17	Tunca et al. (22)	NA	NA	22	+/+/-	-/-	-	-	-	-/-	-	-/-	-/-	-/-/-	-	-	-	-/-/-	-	-	-	-
	c.2639T > C p.L880P	Kancheva et al. (27)	NA	NA	NA	NA	NA	NA	NA	NA	NA	NA	NA	NA	NA	NA	NA	NA	NA	NA	NA	NA	NA
	c.2826G > A p.M942I	Schüle et al. (16)	NA	NA	8	+/+/-	-/+	+	+	-	-/-	+	-/-	-/-	-/-/-	-	-	-	-/-/-	-	-	-	-
			NA	NA	3	+/+/-	-/+	-	-	-	-/-	-	-/-	-/-	-/-/-	-	-	-	-/-/-	+	-	-	-
			NA	NA	6	+/+/-	-/+	-	+	-	-/-	-	-/-	-/-	-/-/-	-	-	-	-/-/-	-	-	-	+
Exon 20	p.Arg1209 fs^*^120	Hanein et al. (19)	M	38	12	+/+/-	+/-	+	+	-	-/-	-	+/-	-/-	-/-/-	-	-	+	-/-/-	+	+	+	+
			F	34	14	+/+/-	+/-	+	+	-	-/-	-	-/-	-/-	-/-/-	-	-	+	-/-/-	+	-	+	+
			M	21	9	+/+/-	+/-	+	+	-	-/-	-	+/+	-/-	-/-/-	-	-	+	-/-/-	+	+	-	-
			M	17	5	+/+/-	-/+	+	-	-	-/-	-	-/-	-/-	-/-/-	-	-	-	-/-/-	+	-	-	-
	c.3118T > A p.S1040T	Schüle et al. (16)	NA	NA	22	+/+/-	-/-	-	+	-	-/-	+	-/+	-/-	-/-/-	-	-	-	-/-/-	-	-	-	-
			NA	NA	11	+/+/-	-/+	-	-	-	-/-	-	-/+	-/-	-/-/-	+	-	-	-/-/-	+	-	+	-
			NA	NA	1	+/+/-	-/+	+	-	-	-/-	+	-/-	+/-	-/-/-	+	+	-	-/-/-	-	+	-	-
	c.3811delT p.S1271 Lfs44	Pashaei et al. (5)	F	20	5	+/+/-	+/-	-	-	-	+/-	-	-/-	-/-	-/-/-	-	-	-	-/-/-	+	+	-	-
Exon 21	c.3935C > A p.S1312^*^	Vantaggiato et al. (17)	NA	27	14	+/+/+	-/-	-	+	-	+/-	+	-/-	+/-	-/-/-	-	+	-	+/-/+	+	+	-	-
			NA	46	14	+/+/+	-/-	+	+	+	+/-	-	+/-	-/-	-/-/+	-	-	-	-/+/-	+	+	-	-
			NA	45	17	+/+/+	-/-	+	+	+	+/-	-	+/-	-/-	-/+/+	-	-	-	+/+/+	+	+	-	-
	c.3417_3418 insTA p.Lys 1140^*^	Renvoisé et al. (25)	F	26	15	+/+/-	+/+	+	-	-	-/-	-	-/-	-/-	-/-/-	+	-	-	-/-/-	+	+	-	-
	c.4068_4069 delTG p.C1356^*^	Goizet et al. (14)	F	38	18	+/+/+	-/-	+	+	+	+/-	-	+/+	-/-	-/-/-	-	-	-	-/-/-	+	-	+	-
			M	34	18	+/+/+	-/-	+	+	+	+/-	-	+/+	-/-	-/-/-	-	-	-	-/-/-	+	-	+	-
	c.4132C > T p.Arg1378^*^	Pensato et al. (18)	M	22	NA	+/+/-	-/-	-	-	-	-/-	-	-/-	-/-	-/-/-	-	-	-	-/-/-	-	-	-	-
	c.4181G > A p.W1394^*^	Lazaridis et al. (28)	NA	NA	NA	NA	NA	NA	NA	NA	NA	NA	NA	NA	NA	NA	NA	NA	NA	NA	NA	NA	NA
	c.4401C > T p.P1467P	Schüle et al. (16)	NA	NA	20	+/+/-	-/-	+	-	-	-/-	+	-/+	-/-	-/-/-	-	-	-	-/-/-	-	-	-	-
	c. 4278 G > A	Dong et al. (29)	M	21	6	+/+/-	+/+	-	-	-	-/+	+	-/-	-/-	-/-/-	-	-	-	-/-/-	+	+	-	+
	c.4312C > T p.Arg1438^*^	Hanein et al. (19)	F	33	13	+/+/+	+/+	-	-	-	-/+	-	+/-	-/-	-/-/-	-	+	-	-/-/-	-	-	+	-
			M	32	14	+/+/+	+/+	+	-	-	-/+	-	+/-	-/-	-/-/-	-	-	-	-/-/-	-	-	-	-
			M	30	16	+/+/+	+/+	-	-	-	-/+	-	+/-	-/-	-/-/-	-	-	-	-/-/-	-	-	-	-
			F	27	12	+/+/-	+/+	-	-	-	-/-	-	-/-	-/-	+/-/-	-	-	-	-/-/-	-	-	-	-
			M	27	12	+/+/-	+/-	-	-	-	-/-	-	-/-	-/-	+/-/-	-	-	-	-/-/-	-	-	-	-
			F	18	16	+/+/-	-/-	-	-	-	-/-	-	-/-	+/-	-/-/-	-	-	-	-/-/-	-	-	-	-
Exon 22	c.4539 delG p.K1514 Sfs^*^26	Koh et al. (30)	F	36	14	+/+/-	+/-	+	-	-	-/-	+	-/-	-/-	-/-/-	-	-	-	-/+/-	+	+	+	-
Exon 25	c.4804C > T p.Arg 1602Ter	Chakrabarty et al. (31)	F	24	4	+/+/-	-/-	-	-	-	-/-	-	-/-	-/-	-/-/-	-	-	-	-/-/-	-	-	-	-
Exon 26	c.5036delT p.L1679 Rfs^*^8	Goizet et al. (14)	M	23	4	+/+/-	+/+	+	-	+	-/-	-	-/-	-/-	-/-/-	-	-	-	-/-/-	+	+	-	-
	c.5203C > T p.Gln 1735^*^	Pensato et al. (18)	NA	NA	NA	NA	NA	NA	NA	NA	NA	NA	NA	NA	NA	NA	NA	NA	NA	NA	NA	NA	NA
	c.5215C > T p.Gln 1739^*^	Yoon et al. (15)	F	32	6	+/+/-	-/-	+	-	-	+/-	-	-/-	-/-	-/-/-	-	-	-	-/-/-	+	+	+	-
Exon 28	c.5415delC p.R1806 Gfs^*^36	Hsu et al. (38)	F	31	9	+/+/-	+/-	-	-	+	+/-	-	-/-	-/-	-/-/-	-	-	-	-/-/-	+	+	-	-
	c.5422C > T p.Q1808^*^	Goizet et al. (14)	M	-	14	+/+/-	+/+	-	-	+	-/-	-	-/-	-/-	-/-/-	-	-	-	-/-/-	+	+	-	-
Intron 28-29	c.5485-1G > A	Hanein et al. (19)	F	31	12	+/+/+	+/+	-	+	-	-/-	-	-/-	+/+	+/-/-	-	-	-	-/-/-	-	-	-	-
			M	30	10	+/+/+	+/-	-	-	-	-/-	-	-/-	+/-	+/-/-	-	-	-	-/-/-	-	-	-	-
			F	25	10	+/+/+	-/-	-	-	-	-/-	-	-/-	-/-	+/-/-	-	-	-	-/-/-	-	-	-	-
			M	16	10	+/+/-	+/-	-	+	-	-/-	-	-/-	+/-	-/-/-	-	-	-	-/-/-	-	-	-	-
	c.5612G > A p.C1871Y	Schüle et al. (16)	NA	NA	16	+/+/-	-/-	+	-	-	-/-	+	-/-	-/-	-/-/-	-	-	-	-/-/-	-	-	+	-
			NA	NA	1	+/+/-	-/+	+	+	-	-/-	+	-/-	-/-	-/-/-	-	-	-	-/-/-	-	-	-	-
Intron 31-32	c.5791–6G > A r.5791_5792 ins5791− 4_5791–1, p.A1931 PfxX1957X	Goizet et al. (14)	M	34	18	+/+/+	-/-	+	+	+	+/-	-	+/+	-/-	-/-/-	-	-	-	-/-/-	+	-	+	-
			F	38	18	+/+/+	-/-	+	+	+	+/-	-	+/+	-/-	-/-/-	-	-	-	-/-/-	+	-	+	-
Exon 32	c.6011G > C p.S2004T	Goizet et al. (14)	M	29	16	+/+/-	+/-	-	-	+	-/-	-	-/-	-/-	-/-/-	-	-	-	-/-/-	+	+	+	+
			F	33	13	+/+/-	+/-	+	-	+	-/-	-	-/-	-/-	-/-/-	-	-	-	-/-/-	-	-	-	-
Exon 34	c.6296 dup p.Asn2100 Glufs^*^12	Mallaret et al. (32)	F	17	16	+/+/-	+/+	+	+	-	-/-	-	-/-	-/-	-/-/-	-	-	-	-/-/-	+	-	-	-
	c.6296_6297 insT p.L2099 Lfs^*^12	Goizet et al. (14)	F	30	14	+/+/+	+/+	+	-	+	-/-	-	-/-	-/-	-/-/-	-	-	-	+/+/-	-	-	-	-
Exon 35	c.6398_6401 delGGGA p.R2133 Asnfs^*^15	Özdemir et al. (33)	F	14	13	+/+/-	+/+	+	-	-	-/-	-	+/-	-/-	-/-/-	-	-	-	-/-/-	-	+	-	+
Exon 36	c.6702_6 771del p.Trp2234 Cysfs^*^2238	Hanein et al. (19)	F	24	18	+/+/+	-/+	-	-	-	-/+	-	-/-	-/-	-/-/-	-	-	-	-/-/-	+	+	+	-
			F	23	19	+/+/+	-/+	-	-	-	-/-	-	-/-	-/-	+/-/-	+	-	-	+/+/-	+	+	-	-
	c.6744_6746 delGAA p.2248 delLys	Pensato et al. (18)	M	25	NA	+/+/-	-/-	-	-	-	-/-	-	-/-	-/-	-/-/-	-	-	-	-/-/-	-	-	-	-
Exon 37	c.6787 delG p.D2263 Tfs^*^7	Jiao et al. (34)	M	24	16	+/+/-	-/-	+	-	-	-/-	-	-/-	-/-	-/-/-	-	-	-	-/-/-	+	+	-	-
	c.6940A > T p.K2314^*^	Denora et al. (21)	F	33	21	+/+/-	-/-	-	-	+	-/-	-	+/-	-/-	-/-/-	-	-	+	-/-/-	-	-	-	-
Exon 38	c.7111dupA p.M2371Nfs^*^ 51	this case 2021	F	19	18	+/+/-	+/+	-	+	+	-/-	-	-/-	-/-	-/-/-	-	-	-	-/-/-	+	-	-	-
Intron 38-39	c.7128 + 1G > C	Schüle et al. (16)	M	19	17	+/+/-	+/+	+	-	-	-/-	-	-/-	-/-	-/-/-	-	-	-	-/-/-	+	+	+	-
Intron 38-39	c.7128 + 2T > A r.6987_ 7128del p.R2329R fsX2337	Goizet et al. (14)	F	31/13	13	+/+/+	-/+	-	-	+	+/-	-	-/+	-/-	-/-/-	+	+	-	-/-/-	-	-	-	-

F, female; M, male; + is positive, - is negative; TCC, Thin corpus callosum; WMH, White matter hyperintensities; NA, no data.

Options available for the treatment of spastic paraplegia are much less than its clinical and genetic types. Rehabilitation therapy and physical therapy are necessary for the maintenance of muscular strength and coordinated movement, and medications such as oral baclofen, intramuscular injections of botulinum toxin, or intrathecal injections of baclofen can relieve spasms. Although HSP has no impact on the lifespan of patients, it can cause serious disability. Genetic diagnosis and symptoms management are important. Early diagnosis and clinical intervention are also helpful to slow disease progression.

## Conclusion

Here, we reported a new homozygous mutation of the *ZFYVE26* gene, c.7111dupA p.(M2371Nfs^*^51) (Exon 38). There have been no previous reports on this genetic locus mutation with HSP. Gene testing plays an important role in the diagnosis of HSP, and family genetic lineage reveals the source of the pathogenic gene. With the rapid improvement of gene testing technology, the number of known HSP disease-causing gene is increasing, which brings a challenge to the early diagnosis and clinical evolution.

## Data availability statement

The raw data supporting the conclusions of this article will be made available by the authors, without undue reservation.

## Ethics statement

Written informed consent was obtained from the participant/patient(s) for the publication of this case report.

## Author contributions

Z-hL wrote this manuscript. X-yL and Y-yS collected clinical data and references. H-yZ and L-lZ guided the writing and revision of this manuscript. All authors contributed to the article and approved the submitted version.
