# Supplementary material for: Case report: Hereditary spastic paraplegia with a novel homozygous mutation in ZFYVE26
Source: Front Neurol. 2023 Aug 23;14:1160110. doi: 10.3389/fneur.2023.1160110 (PMC10482258; doi:10.3389/fneur.2023.1160110)
Supplement: Supplementary file 1 [file Presentation_1.PDF]

韦翰斯生物

基因检测报告

GENETIC TEST REPORT

姓　　名： 王迎杰

送检项目： 家系全外显子组测序（含线粒体）

报告时间： 2021 年 05 月 14 日

报告编号： 2021WES66011

注意：此报告结果仅对本次受检样本负责，限受检者本人拆阅。

检测结果仅供参考，具体方案请咨询临床医生。

如果对检验结果有疑问，请于收到报告后7个工作日内与我们联系，谢谢合作。

上海韦翰斯生物医药科技有限公司

此页为空白页。

基本信息

姓名：王迎杰　　样本类型：血液　　送检单位：上海瑞金医院

性别：女　　送样日期：2021/03/11　　送检科室：神内

年龄：19岁　　录样日期：2021/04/15　　送检医师：周医生

民族：/　　报告日期：2021/05/14　　样本编号：210066011

临床信息：受检者SMA？无相关家族史。

家属信息

亲缘关系：受检者父亲　　样本类型：血液　　样本编号：210069160

亲缘关系：受检者母亲　　样本类型：血液　　样本编号：210069161

检测结果

阳性

检出与受检者临床表型相关或部分相关的基因变异

1. 临床表型相关基因变异

发现受检者 *ZFYVE26* 基因上的 1 个纯合移码变异：c.7111dupA:p.M2371Nfs*51。二代测序结果显示该变异遗传自受检者父母，受检者父母在该位点均为杂合变异。*ZFYVE26* 基因异常导致常染色体隐性遗传的痉挛性截瘫 15 型（SPASTIC PARAPLEGIA 15, AUTOSOMAL RECESSIVE; SPG15; OMIM#270700）（详见疾病描述）。请结合受检者的临床症状、家族史以及其他检查结果进一步确定该变异与临床表型的相关性。

基因	染色体位置（hg19）	dbSNP ID	变异命名	gnomAD_EAS 人群频率	ACMG 变异评级	合子类型	亲属检测结果	
							父亲	母亲
ZFYVE26	chr14:68220805	/	ZFYVE26:NM_015346:exon38:c.7111dupA:p.M2371Nfs*51	未收录	致病	纯合	杂合	杂合

2. 次要发现

无次要发现。

基因	染色体位置（hg19）	dbSNP ID	变异命名	gnomAD_EAS 人群频率	ACMG 变异评级	合子类型	亲属检测结果	
							父亲	母亲
/	/	/	/	/	/	/	/	/

备注：次要发现（secondary findings）仅限美国医学遗传和基因组学会（ACMG）政策声明（ACMG SF v2.0）[1]的 59 个基因中的已知致病（known pathogenic, KP）和/或预期致病（expected pathogenic, EP）变异。

3. 线粒体基因组检测结果

线粒体基因组上未发现与受检者临床表型高度相关的致病/可能致病变异。

基因	基因组位置（hg19）	dbSNP ID	变异命名	变异类型	变异频率	变异分类	亲属检测结果	
							父亲	母亲
/	/	/	/	/	/	/	/	/

遗传咨询和建议：

1. *ZFYVE26* 基因变异导致的痉挛性截瘫 15 型以常染色体隐性方式遗传。对于常染色体隐性遗传疾病来说，单个致病性变异的携带者并不会发展为患者，只有纯合致病性变异或复合杂合型致病性变异（反式）才会导致疾病的发生。若父母均为携带者，每次生育的子女均有 25%的可能为患者。建议根据该检测结果，对受检者进行进一步临床检查。

2. 建议结合受检者家族临床表型和基因型进一步分析确定该变异的临床意义。建议受 检者与主治医生共同制定进一步治疗和生育管理方案。建议遗传咨询。

报告人：那晓雪　　审核人：李雯雯　　盖章：上海韦翰斯生物医药科技有限公司 报告专用章

结果解释

1. 临床表型相关基因变异

ZFYVE26:NM_015346:exon38:c.7111dupA:p.M2371Nfs*51

1.1 疾病描述与遗传特点[4-6]

遗传性痉挛性截瘫（hereditary spastic paraplegia, HSP）是一组具有显著临床和遗传学异质性的神经退行性疾病，主要的临床特征表现为双下肢缓慢进行性无力和痉挛性截瘫，发病率约为 1.8/100,000。常见的病理改变为皮质脊髓束及后柱最长的下行性运动纤维远端轴索的变性，伴或不伴脱髓鞘改变，主要由囊泡转运、轴索运输、细胞器形成、线粒体功能异常、脂质代谢障碍、髓鞘异常等分子机制所致。根据遗传方式可分为：常染色体显性遗传、常染色体隐性遗传、X 连锁隐性遗传和线粒体母系遗传。随着分子遗传学技术的发展，迄今已报道了 78 个致病基因相关位点，59 个致病基因被克隆。

ZFYVE26 基因位于 14q24.1。*ZFYVE26* 基因异常导致常染色体隐性遗传的痉挛性截瘫 15 型（OMIM#270700），该疾病主要临床症状包括尿急、尿失禁、视力障碍、视网膜变性、黄斑变性、眼球震颤、情绪波动、智力障碍、共济失调、痉挛性截瘫、构音障碍、弓形足、下肢痉挛、胼胝体发育不全、阵挛、大便失禁、膀胱括约肌功能障碍、周围神经轴突神经变性、远端肌萎缩、下肢肌无力等。

1.2 变异解释

发现受检者 *ZFYVE26* 基因上的 1 个纯合移码变异：c.7111dupA:p.M2371Nfs*51，该变异为 cDNA 的第 7111 位碱基重复，导致 *ZFYVE26* 基因第 2371 位密码子处发生移码，可能导致基因功能缺失或部分缺失。二代测序结果显示该变异遗传自受检者父母，受检者父母在该位点均为杂合变异。家系图如下所示：

标志	关系	家系图
I-1	父亲	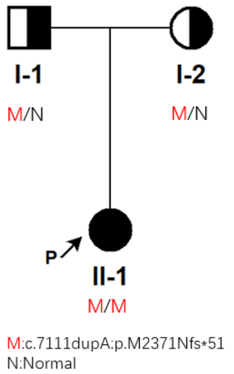
I-2	母亲	
II-1	受检者	

该变异目前尚无文献报道。参照美国医学遗传学和基因组学学会（American College of Medical Genetics and Genomics, ACMG）相关指南[2]，该变异为致病性变异。分类依据如下表：

变异：ZFYVE26:NM_015346:exon38:c.7111dupA:p.M2371Nfs*51

ACMG 证据	证据描述	参考文献	分类结果
**PVS1**	*ZFYVE26* 基因致病机制为功能缺失（LOF），该变异为移码变异，可能导致蛋白质功能缺失。	[7]	致病
**PM2_Supporting**	该变异为罕见变异，在 gnomAD 数据库东亚普通人群中未收录。	/	
**PP4**	该变异引起的疾病符合单基因遗传病痉挛性截瘫 15 型。	/	
**PM3_Supporting**	本次检测在受检者中发现该变异的纯合子。	/	

附录信息-变异发现

变异：ZFYVE26:NM_015346:exon38:c.7111dupA:p.M2371Nfs*51

受检者

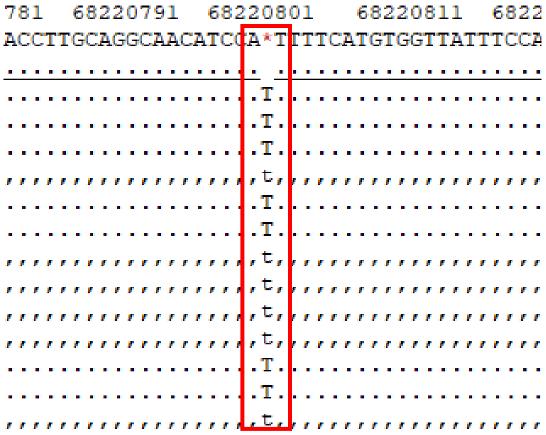

受检者父亲

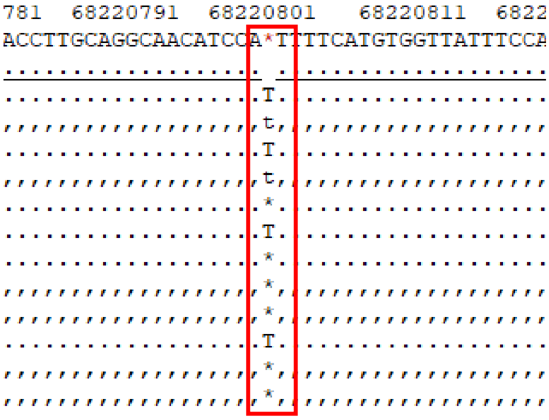

受检者母亲

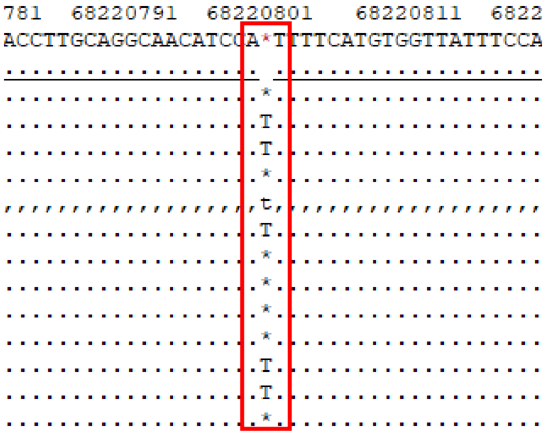

附录信息-其他变异

下表变异仅与受检者部分临床表型相关/不符合遗传方式/在正常人群中频率较高/致病性尚不明确等，仅供临床医生参考。

变异编号	基因名	染色体位置（hg19）	dbSNP ID	变异命名	gnomAD_EAS人群频率	ACMG 变异评级	相关疾病 (OMIM,遗传方式)	合子类型	亲属检测结果	
									父亲	母亲
1	*CASQ1*	chr1:160160588	rs557844880	CASQ1:NM_001231:exon1:c.47G>A:p.R16Q	0.0000544	意义不明	1、CASQ1 聚积性空泡性肌病 (616231, AD)	杂合	杂合	未检出
2	*CPT1C*	chr19:50204029	rs763244007	CPT1C:NM_001136052:exon5:c.370G>A:p.V124M	0	意义不明	1、痉挛性截瘫 73 型常染色体显性遗传(616282, AD)	杂合	未检出	杂合

变异1：

*CASQ1*基因异常可导致常染色体显性遗传的CASQ1聚积性空泡性肌病（OMIM#616231），该病临床表现有肌肉痉挛、肌痛、近端肌无力、上和/或下肢可能受累、易疲劳、肌纤维坏死、血清肌酸激酶升高、CASQ1聚积性空泡性肌病等[8]。

经检测和分析，在受检者 *CASQ1* 基因中发现一个杂合错义变异。该变异在 gnomAD 普通人群数据库东亚人群中的频率为 0.0000544。该变异目前尚无文献报道。仅有 1 个生信计算方法预测该变异可对基因或基因产物造成有害影响。根据 ACMG 相关指南[2]，该变异为意义不明变异。二代测序结果显示该变异遗传自受检者父亲。该病仅与受检者临床表现部分相关，请结合受检者的临床症状、家族史以及其他检查结果进一步确定该变异与临床表型的相关性。

变异2：

*CPT1C*基因异常可导致常染色体显性遗传的痉挛性截瘫73型（OMIM#616282），该病临床表现有轻度排尿功能障碍、足畸形、轻度的近端肌无力、肌萎缩、痉挛性截瘫、行走困难、反射亢进等[9]。

经检测和分析，在受检者 *CPT1C* 基因中发现一个杂合错义变异。该变异在 gnomAD 普通人群数据库东亚人群中的频率为 0。该变异目前尚无文献报道。有 4 个生信计算方法预测该变异可对基因或基因产物造成有害影响。根据 ACMG 相关指南[2]，该变异为意义不明变异。二代测序结果显示该变异遗传自受检者母亲。该病仅与受检者临床表现部分相关，请结合受检者的临床症状、家族史以及其他检查结果进一步确定该变异与临床表型的相关性。

备注：“意义不明变异”是指基于目前医学进展和变异分类证据，该变异的致病性尚不明确。ACMG 建议不宜将意义不明变异用于临床决策，需结合临床进行评估。此类变异主要包括以下几种情况：（1）目前没有报道或数据库收录，或者研究较少，支持致病或支持良性的证据不充分；（2）目前支持致病和支持良性的证据均存在。随着医学研究的发展，意义不明变异分类可能会发生改变。

附录信息-检测项目

家系全外显子组测序（含线粒体）

附录信息-检测方法

1. 测序：从受检者样本中提取基因组 DNA，构建基因组文库。通过探针杂交捕获目标基因（约 20,000 个）外显子及毗邻剪接区域（约 20bp），以及线粒体基因组全长，并进行富集。对富集的基因进行质量控制，利用高通量测序仪进行测序。本次受检样本目标区域捕获测序参数为：

	受检者	受检者父亲	受检者母亲
样本编号	210066011	210069160	210069161
原始测序数据量（Mb）	10,485.6	10,558.79	11,268.36
测序总 reads 数（条）	104,856,020	105,587,862	112,683,604
目标区域平均测序深度（X）	140.14	132.63	141.58
目标区域平均测序深度≥1X 覆盖度（%）	99.8	99.88	99.83
目标区域平均测序深度≥10X 覆盖度（%）	99.62	99.8	99.64
目标区域平均测序深度≥30X 覆盖度（%）	99.31	99.33	99.36
20%X 平均深度覆盖度（%）	99.46	99.53	99.44
线粒体基因组平均测序深度（X）	2,502.07	3,100.36	3,920.80
Q30 合格率（%）	95.62	93.90	92.38

2. 分析：测序原始数据首先去除不符合质控要求的 reads，然后运用 BWA 软件与 UCSC 提供的 hg19 版本人类基因组参考序列进行比对，经过 GATK 的 HaplotypeCaller 找出其中的 SNV 和 InDel 变异，再经过下列专业数据库和生信预测软件，以及韦翰斯自有的本地数据库和分析软件进行进一步注释和筛选。采用 xhmm 和 clamms 算法对探针覆盖区域进行拷贝数变异分析。

   人群变异频率数据库：1000 Genomes、ESP、ExAC、gnomAD 等；
   位点及疾病数据库：dbSNP、OMIM、HGMD、ClinVar、Decipher、DGV 等；
   生信预测软件：SIFT、Polyphen2、 LRT、MutationTaster、FATHMM、M-CAP、CADD、REVEL、dbscSNV、SpliceAI 等。

3. 解读：序列变异数据解读规则参考美国医学遗传学和基因组学学会（American College of Medical Genetics and Genomics, ACMG）遗传变异分类标准与指南[2]，以及 ClinGen 序列变异解读（SVI）专家组陆续发布的一系列通用建议和细则（例如 PVS1、PS2/PM6、PS3、BA1、PM3、PP5 和 BP6 等），以及针对特定基因（例如 *PTEN*、*CDH1*、*PAH* 等）和疾病（如 Rasopathy 和耳聋等）的解读指南，进行进一步的细化解读。拷贝数变异(CNV)解读规则参考 2019 版 ACMG 拷贝数变异解读和报告指南[3]。

根据受检者的临床表型和家族史进行分析，针对已知的、明确的与遗传病相关的基因进行分析，一些功能和致病性尚未明确的基因不在本次分析范围内。除非有相关致病性的文献报道或者数据库收录，否则序列变异分析将不包括常见的良性多态性变异，以及不影响 mRNA 剪接的同义变异和内含子变异。如未发现与受检者临床表型相关的拷贝数变异则不报告。

附录信息-检测局限

1. 本次检测报告内容仅限于能解释或部分解释受检者相关表型的致病/可能致病/意义不明变异，以及 ACMG 政策声明（ACMG SF v2.0）[1]的 59 个基因中的已知致病（known pathogenic, KP）和/或预期致病（expected pathogenic, EP）变异，其他变异不在本报告范围之内。

2. 此检测方法适用于检测探针覆盖的基因外显子及毗邻剪接区域(约 20bp)的单碱基变异、小片段的插入缺失变异（30bp 以内），可对探针覆盖范围内的拷贝数变异（超过 3 个连续的外显子）进行分析，但非常规拷贝数变异检测方法，对其检出能力有限，且存在一定程度的假阴性或者假阳性，建议对报告提示的与临床表型高度相关的拷贝数变异进行 MLPA、qPCR、CNV-seq、基因芯片等方法验证。

3. 此检测方法不适用于检测探针未覆盖区域的变异（如调控区及深度内含子区的变异），基因组的特殊结构变异（如倒位、异位、重排、单亲二体等），动态突变，极低比例的嵌合变异，以及表观遗传变异等。本检测所用的 DNA 来源于受检者血液或其他体细胞，非源自生殖细胞，故不能排除生殖细胞嵌合现象所致的解读偏差。

4. 由于部分基因存在高重复低复杂度区域，或同源序列，或高度同源的假基因，或 GC 含量较高，这些区域在检测时可能低覆盖或无覆盖，检出能力有限。在数据分析时，为保证分析精确，测序质量过低的变异已被过滤，但总体覆盖度一般可达 95%以上。

5. 此检测方法不适用于检测线粒体基因组的大片段缺失/重复，对线粒体基因组极低比例的变异检出能力有限。且由于线粒体基因组具有组织异质性，本检测结果仅能反映本次受检样本中线粒体基因组的情况。

6. 受限于基因检测技术和目前对遗传病的认识水平，如未检出能够解释受检者临床表型的特定基因及致病变异位点（即阴性结果），也并不能完全排除受检者存在某种遗传病的可能性。因为某些遗传病的发病可能与未知基因、检测未覆盖的区域的变异，或无法检出的变异类型有关，也可能由于疾病本身的复杂性，遗传因素并非受检者发病的主导原因。即使基因检测结果为阴性，仍建议以医生诊断、其他临床检测和家族史为准。

7. 若受检者提供的送检信息不全或不实，可能会导致分析结果存在偏差。

8. 如临床高度怀疑某一基因或者某种遗传病，但检测结果为阴性，可考虑其他更有针对性的检测方法。

9. 所有结果注释、解读均依赖于临床提供的病史信息，并基于目前的相关研究证据（包括现有的数据库信息和已发表的文献资料），随着研究的深入，对基因变异的解读可能会发生改变。如受检者出现新的临床表型或者收集到更详细的家族史，可与本公司联系重新分析数据或解读咨询。

附录信息-参考文献

[1] Kalia SS., Adelman K., Bale S.J., *et al.* Recommendations for reporting of secondary findings in clinical exome and genome sequencing, 2016 update (ACMG SF v2.0): a policy statement of the American College of Medical Genetics and Genomics. Genet Med,2017. 19: 249-255.

[2] Richards, S., et al. Standards and guidelines for the interpretation of sequence variants: a joint consensus recommendation of the American College of Medical Genetics and Genomics and the Association for Molecular Pathology. Genet Med, 2015. 17(5): 405-424.

[3] Riggs E R, Andersen E F, Cherry A M, et al. Technical standards for the interpretation and reporting of constitutional copy-number variants: a joint consensus recommendation of the American College of Medical Genetics and Genomics (ACMG) and the Clinical Genome Resource (ClinGen)[J]. Genetics in Medicine, 2020, 22(2): 245-257.

[4] OMIM, https://omim.org/entry/270700.

[5] 詹飞霞, 曹立. 遗传性痉挛性截瘫4型发病机制的研究进展[J]. 上海交通大学学报(医学版), 2017, 037(009):1281-1285.

[6] 张超. 遗传性痉挛性截瘫4型临床表型及SPAST基因新突变和蛋白功能学研究. 安徽理工大学.

[7] Hanein Sylvain,Martin Elodie,Boukhris Amir et al. Identification of the SPG15 gene, encoding spastizin, as a frequent cause of complicated autosomal-recessive spastic paraplegia, including Kjellin syndrome.[J] .Am J Hum Genet, 2008, 82: 992-1002.

[8] OMIM, https://omim.org/entry/616231.

[9] OMIM, https://omim.org/entry/616282.

附录信息-专业名词解释

1. **染色体：**分为常染色体和性染色体。人类有 22 对常染色体和 1 对性染色体（X/Y），一共 46 条。父母每一方各遗传 23 条染色体（22 条常染色体+1 条性染色体）给他们的后代。正常男性染色体核型为 46,XY，正常女性染色体核型为 46,XX。

2. **基因：**染色体上具有遗传效应的 DNA 片段。

3. **遗传方式：**遗传病所呈现的孟德尔遗传方式，分为常染色体显性遗传（AD）、常染色体隐性遗传（AR）、X 连锁显性遗传（XLD）、X 连锁隐性遗传（XLR）、Y 染色体遗传、母系遗传等，一种疾病有可能有多种遗传方式。

4. **合子类型**

   杂合子（Heterozygous）：一对等位基因中仅一个拷贝发生了变异。
   纯合子（Homozygous）：一对等位基因中两个拷贝均发生了变异。
   半合子（Hemizygous）：男性 X 染色体上发生了变异。

5. **rs 号：**变异在 dbSNP 数据库的编号，一个变异有无 rs 号与是否致病无直接关系，无 rs 号提示有可能为新发现的变异位点。

6. **MAF（Minor Allele Frequency）：**最小等位基因频率，通常是指在给定人群中的不常见的等位基因发生频率，例如 TT，TC，CC 三个基因型，在人群中 C 的频率=0.36，T 的频率=0.64，则等位基因 C 就为最小等位基因频率，MAF=0.36。位点的人群频率可以用来区别常见变异和罕见变异，MAF 越小，说明是罕见变异；

7. **人群频率数据库：**此类数据库一般基于大规模人群测序数据，提供单基因遗传病人群频率查询。对于评估罕见位点的致病性，如果某位点在此类数据库未见报道或频率极低，则致病几率增大，如 1000 Genomes、ESP、ExAC、gnomAD 等，但并不是说一定致病，是否为致病变异需综合多方面证据评估。本报告表格中提示的人群频率主要参考 gnomAD 东亚人群。

8. **生信预测软件：**基于保守性原理或区域特殊性，通过计算机预测氨基酸变化对蛋白功能造成的影响是有害还是可容忍。如 SIFT、Polyphen2、 LRT、MutationTaster、FATHMM、M-CAP、CADD、REVEL、dbscSNV 等。

9. **变异命名：**根据人类基因组变异协会（Human Genome Variation Society, HGVS）提出的命名规则，包括转录本编号（以前缀“NM_”表示），cDNA 序列 （以前缀“c.”表示），氨基酸序列（以前缀 “p.”表示）。

   cDNA 中一种碱基被另一种碱基取代，以 “>”进行表示，如 c.1529A>G，表示与参考序列相比，第 1529 位的腺嘌呤（A）被鸟嘌呤（G）所取代；氨基酸序列中 p.Q510R，表示第 510 位的 Q（Glutamine 谷氨酰胺）被 R（Arginine 精氨酸）取代，即错义变异。

缺失以“del”进行表示；cDNA 序列如：c.2052delA，表示与参考序列相比，第 2052 位发生 A 的缺失；氨基酸序列中如 p.A3_S5del，表示氨基酸序列中从第 3 位的 A 到第 5 位的 S 发生了缺失；

插入以“ins”进行表示，cDNA 序列如：c.5756_5757insAGG，表示与参考序列相比，在第 5756 与 5757 位点之间插入了三个碱基 AGG；氨基酸序列中如 p.L2_Q3insS，表示在第 2 位的 L 和第 3 位的 Q 之间插入了 S；

重复以“dup”进行表示；cDNA 序列如：c.6_8dupT，表示从第 6 位到第 8 位发生了 T 的重复；氨基酸序列中如 p.A2[10]，表示第 2 位的 A 重复了 10 次；

10. **变异评级：**根据 ACMG 指南对变异进行的分级，分别为：致病（Pathogenic, P）、疑似致病（Likely Pathogenic, LP）、意义不明（uncertain significance, VUS）、疑似良性（Likely Benign, LB）、良性（Benign, B）。

11. **次要发现：**次要发现（Incidental findings or secondary findings）指的是临床基因检测时（特别是全外显子和全基因组检测），在与受检者临床表现不相关的基因中发现的致病和疑似致病变异。ACMG 官方制定的次要发现基因列表（v2.0）共包含 59 个基因，选择这些基因主要是因为这些基因对应的疾病有可靠的诊断方法，且存在有效的干预或治疗办法。ACMG 建议在开展全外显子或者全基因组检测时报告次要发现。次要发现基因列表如下：

BRCA1	*MSH6*	*VHL*	*SDHAF2*	*NF2*	*ACTA2*	*TPM1*	*LMNA*	*DSG2*	*PCSK9*
BRCA2	*PMS2*	*MEN1*	*SDHC*	*COL3A1*	*MYH11*	*MYL3*	*RYR2*	*KCNQ1*	*ATP7B*
TP53	*APC*	*RET*	*SDHB*	*FBN1*	*MYBPC3*	*ACTC1*	*PKP2*	*KCNH2*	*OTC*
STK11	*MUTYH*	*PTEN*	*TSC1*	*TGFBR1*	*MYH7*	*PRKAG2*	*DSP*	*SCN5A*	*RYR1*
MLH1	*BMPR1A*	*RB1*	*TSC2*	*TGFBR2*	*TNNT2*	*GLA*	*DSC2*	*LDLR*	*CACNA1S*
MSH2	*SMAD4*	*SDHD*	*WT1*	*SMAD3*	*TNNI3*	*MYL2*	*TMEM43*	*APOB*	
