# Supplementary material for: Case report: Hereditary spastic paraplegia with a novel homozygous mutation in ZFYVE26
Source: Front Neurol. 2023 Aug 23;14:1160110. doi: 10.3389/fneur.2023.1160110 (PMC10482258; doi:10.3389/fneur.2023.1160110)
Supplement: Supplementary file 2 [file Presentation_2.PDF]

# 发布病例报告知情同意书

敬爱的患者，您好：

经住院后有关检查，当前考虑您的遗传性痉挛性截瘫致病基因突变为新突变。此种基因位点突变国际上暂未见报道，属于新的突变类型，我们希望发布有关内容，加强医师对该疾病的认识。发布的有关文章内会隐去您的个人信息，但会交代整个发病过程及治疗转归。假定你同意，可签订次知情同意书。

意见：同意

患者签名：王迎杰

医生签名：曾丽莉

日　　期：2021 年 5 月 16 日
