# Supplementary material for: Case report: Hereditary spastic paraplegia with a novel homozygous mutation in ZFYVE26
Source: Front Neurol. 2023 Aug 23;14:1160110. doi: 10.3389/fneur.2023.1160110 (PMC10482258; doi:10.3389/fneur.2023.1160110)

Supplement 2. The sequence of the novel pathogenic mutation in the gene *ZFYVE26.*

Patienet


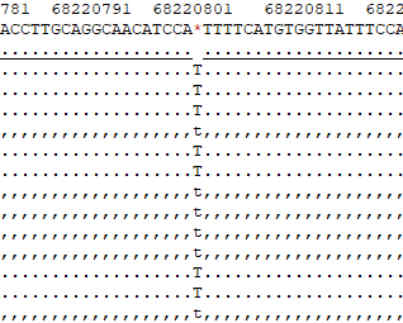


Father


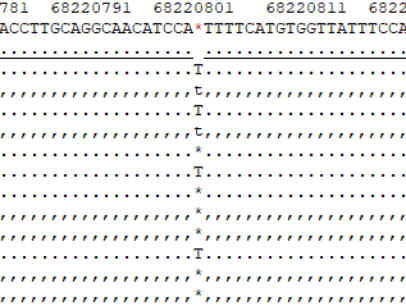


Mother


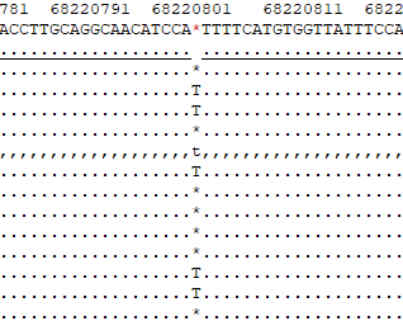

Supplement: Supplementary file 4 [file Data_Sheet_2.docx]
