# Supplementary material for: Case report: Hereditary spastic paraplegia with a novel homozygous mutation in ZFYVE26
Source: Front Neurol. 2023 Aug 23;14:1160110. doi: 10.3389/fneur.2023.1160110 (PMC10482258; doi:10.3389/fneur.2023.1160110)
Supplement: Supplementary file 5 [file Data_Sheet_3.docx]

Supplement 3. Other mutations detected

| Gene | Location of chromosomes | dbSNPID | Variant nomenclature | Patient | Father | Mother |
| --- | --- | --- | --- | --- | --- | --- |
| *CASQ1* | chr1:160160588 | rs557844880 | *CASQ1*:NM_001231:exon1:c.47G>A:p.R16Q | heterozygosis | heterozygosis | NO |
| *CPT1C* | chr19:50204029 | rs763244007 | *CASQ1*:NM_001231:exon1:c.47G>A:p.R16Q | heterozygosis | NO | heterozygosis |
